# Sikokianin D, A New C-3/C-3"-Biflavanone from the Roots of *Wikstroemia indica*

**DOI:** 10.3390/molecules17077792

**Published:** 2012-06-26

**Authors:** Jie Li, Lin-Yan Lu, Ling-Hui Zeng, Chong Zhang, Jia-Lei Hu, Xiang-Rong Li

**Affiliations:** School of Medicine, Zhejiang University City College, No. 48 Huzhou Road, Hangzhou 310015, Zhejiang, China

**Keywords:** *Wikstroemia indica*, sikokianin D, C-3/C-3"-biflavanone

## Abstract

A new 3,3′′-biflavanone, sikokianin D (**1**), was isolated from the roots of *Wikstroemia indica*, together with two known compounds. Their structures were elucidated by chemical evidence and spectral analyses, including HR-ESI-MS, and 1D- and 2D-NMR techniques.

## 1. Introduction

**Figure 1:**
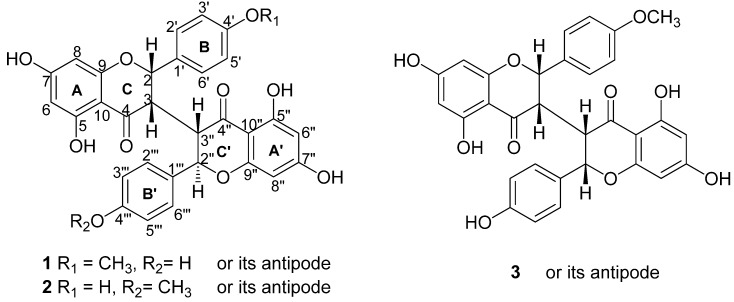
Chemical Structures of **1–3**.

*Wikstroemia indica* (Linn.) C. A. Mey., a shrub of the Thymelaeaceae family, is wildely distributed in the southeast of China. Known as Liaogewang, it has long been used as a folk medicine in southern China for treating arthritis, tuberculosis, syphilis and pertussis [1]. Moreover, *W. indica* has antifungal, anti-inflammatory, anti-cancer, antiviral and antimalarial effects [2,3,4,5,6,7]. The chemical constituents of the roots have been investigated previously, leading to the identification of groups of flavonoid, coumarin and lignan compounds [2,3,4,5,6,7,8,9]. In previous paper [10], we have reported several C-3/C-3"-biflavanones from the roots of *Stellera chamaejasme* L. (Thymelaeaceae) collected in Yunnan. C-3/C-3"-Biflavanones have been shown to exhibit a wide range of pharmacological activities, such as antibacterial, anti-inflammatory, antimalarial, and antitumor activities [4,11,12,13,14]. In connection with these interesting biflavanones, we examined the chemical constituents of other Thymelaeaceae plants and one new C-3/C-3"-biflavanone, sikokianin D (**1**), together with two known compounds, namely sikokianin B (**2**) and sikokianin A (**3**) (Figure 1) was isolated from the roots of *Wikstroemia indica*. This paper describes the isolation and structure elucidation of these compounds.

## 2. Results and Discussion

**Figure 2:**
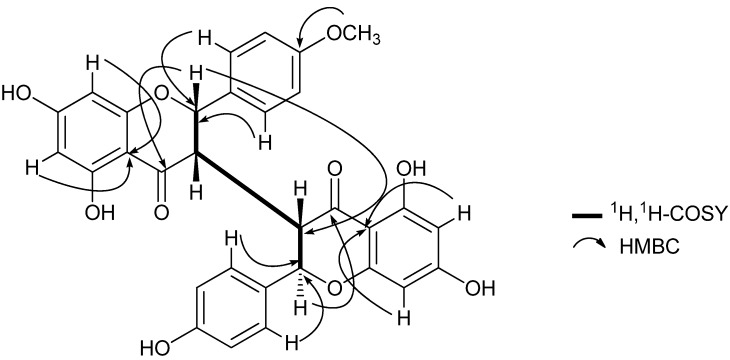
Key ^1^H-^1^H COSY and HMBC correlations of **1**.

Compound **1** was obtained as a pale yellow amorphous powder with optical activity ([α]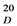: + 231). The HR-ESI-MS of **1** exhibited a quasi-molecular-ion peak ([M+H]^+^) at *m/z* 557.1442 (calc. 557.1448), corresponding to the molecular formula C_31_H_24_O_10_. Moreover, this compound showed positive reaction with HCl-Mg reagent, indicating that it is a flavonoid. The ^1^H-NMR spectrum of 1 (Table 1) displayed signals of one methoxyl group (δ_H_ 3.79, s, 3H), two H-atoms corresponding to H-2 (δ_H_ 5.57, 1H, d, *J* = 5.0 Hz) and H-2" (δ_H_ 5.19, 1H, d, *J* = 9.5 Hz), and two H-atoms corresponding to H-3 (δ_H_ 3.19, 1H, br s) and H-3" (δ_H_ 3.26, 1H, dd, *J* = 9.5, 3.0 Hz) at the rings C and C' of the biflavanone. In the ^1^H- and ^13^C-NMR established by ^1^H-^1^H COSY and HMQC experiments (Table 1), the spectra showed its structural fragments to include two sets of typical 5,7-dioxygenated A rings (δ_H_ 5.74, 5.77, each 1H, d, *J* = 2.0 Hz; δ_H_ 5.78, 5.98, each 1H, d, *J* = 2.0 Hz), and two sets of *para*-oxygenated B rings (δ_H_ 7.22, 6.90, each 2H, d, *J* = 8.5 Hz; δ_H_ 6.93, 6.63, each 2H, d, *J* = 8.5 Hz). From the ^13^C-NMR data (Table 1), two carbonyl groups (δ_C_ 198.5, 196.1) were also observed. These structural fragments were connected to form the given carbon framework of **1** as a dimer of flavanonol derivatives. The partial (-CH-CH-CH-CH-) structure inferred from the ^1^H-^1^H COSY spectrum (bold line in Figure 2) suggested that the linkage of the two flavanones was possible only at the C-3 and C-3" positions, which was supported by the comparison of the ^1^H- and ^13^C-NMR data of **1** with those of known 3,3"-biflavanones [4,6,8,10], and further confirmed by the HMBC correlations of H-2 (δ_H_ 5.57) with C-3" (δ_C_ 51.0). The B ring could be located at C-2, based on the observation of the clear cross-peaks of H-2' and H-6' (δ_H_ 7.22) with C-2 (δ_C_ 81.2). In the same way, linkage of the B' ring to C-2" of the C' ring was deduced by the correlations of H-2"'and H-6"'(δ_H_ 6.93) with C-2" (δ_C_ 83.3). The HMBC cross-peak between the methoxyl group and C-4' on the B ring indicated that the methoxyl group was connected to C-4'.

The stereochemistry at the C-2/C-3 and C-2"/C-3" positions in **1** was determined as *cis-trans* by comparison of the *J* values (*J*_H-2_ = 5.0 Hz and *J*_H-2"_ = 9.5 Hz) with those of the known 3,3"-biflavanones. The key NOESY correlations between H-2" (δ_H_ 5.19) with H-2'(H-6') (δ_H_ 7.22) further confirmed the conclusion above. The relative stereochemistry of compound **1** was confirmed as shown in Figure 1 and the compound named sikokianin D.

Compound **2** was first reported as sikokianin B of which the location of MeO group was unsettled [8], and the exact configuration was elucidated by Nunome [4]. Sikokianin B and sikokianin C were determined by comparing their ^1^H- and ^13^C-NMR and MS data with published values.

**Table 1:** NMR data of sikokianin D (**1**) in CD_3_OD (500 MHz for ^1^H, 125 MHz for ^13^C).

No.	δ_H_ Mult (J = Hz)	δ_C_
2	5.57 d (5.0)	81.2 d
3	3.19 br s	49.3 d
4	-	198.5 s
5	-	165.0 s
6	5.74 d (2.0)	96.0 d
7	-	168.1 s
8	5.77 d (2.0)	97.0 d
9	-	165.0 s
10	-	103.6 s
1'	-	130.0 s
2'	7.22 d (8.5)	128.4 d
3'	6.90 d (8.5)	114.9 d
4'	-	160.9 s
5'	6.90 d (8.5)	114.9 d
6'	7.22 d (8.5)	128.4 d
2''	5.19 d (9.5)	83.3 d
3''	3.26 dd (9.5, 3.0)	51.0 d
4''	-	196.1 s
5''	-	165.3 s
6''	5.78 d (2.0)	97.0 d
7''	-	167.9 s
8''	5.98 d (2.0)	96.4 d
9''	-	163.9 s
10''	-	105.1 s
1'''	-	128.9 s
2'''	6.93 d (8.5)	130.3 d
3'''	6.63 d (8.5)	116.1 d
4'''	-	158.9 s
5'''	6.63 d (8.5)	116.1 d
6'''	6.93 d (8.5)	130.3 d
4'-OCH_3_	3.79 s	55.7 q

## 3. Experimental

### 3.1. General

Melting points were measured on a Thermal Values analytical microscope and are uncorrected. Optical rotations were recorded on a Perkin-Elmer 341 polarimeter. IR spectra were recorded on a Nicolet FI-IR 200SXY spectrophotomer. The spectra of high resolution-electrospray ionization-mass spectrometry (HR-ESI-MS) were acquired with a Micromass Q-TOF mass spectrometer (Waters Corporation USA). ^1^H- and ^13^C-NMR spectra were measured in CD_3_OD with TMS as the internal standard on a Bruker DMX-500 NMR instrument. Silica gel G_254_ and H (Qingdao Sea Chemical Factory, China) were used for TLC and column chromatography, respectively.

### 3.2. Plant Material

The roots of *Wikstroemia indica* were purchased from a Chinese medicine pharmacy in Guangzhou, China, in September, 2011. The authentication process was carried out by Le Cai (Yunnan University). A voucher specimen was deposited in the Zhejiang University City College.

### 3.3. Extraction and Isolation

Air-dried powder roots (2.6 kg) of *W. indica* were extracted exhaustively with 95% aq. EtOH (9 L × 3) at r. t. After concentration *in vacuo*, a crude extract (270 g) was obtained, which was suspended in 1 L H_2_O, and the suspension was extracted successively with petroleum ether (PE, 1 L × 3), EtOAc (1 L × 3), and BuOH (1 L × 3) to yield 34, 110, 89 g fractions, resp. The EtOAc extract was subjected to CC with PE/EtOAc gradient system of increasing polarity (9/1→5/5, 3600 mL) to give five fractions (Fraction 1–5). Fraction 3 was chromatographed repeatedly over SiO2 column with MeOH/H_2_O (7/3→9/1, 1,200 mL) to afford **3** (15 mg). Fraction 4 was subjected to MPLC on octadecyl silica gel (3.5 × 30 cm) eluting by gradient elution with MeOH-H_2_O (5 mL/min, linear gradient, 50:50→90:10) to yield compounds **1** (28 mg) and **2** (36 mg).

*Sikokianin* D (**1**). Yellow amorphous powder, mp 213–215 °C; ([α]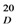: +231 (c = 0.48, MeOH); IR (KBr, cm^−1^): 3362, 1643; ^1^H-NMR and ^13^C-NMR data, see Table 1; HR-ESI-MS: *m/z* 557.1442 [M+H]^+^, calcd for C_31_H_25_O_10_, 557.1448.

*Sikokianin* B (**2**). Yellow amorphous powder. ^1^H-NMR: δ_H_ 3.23 (1H, t, *J* = 3.5 Hz, H-3), 3.33 (1H, dd, *J* = 9.5, 3.0 Hz, H-3"), 3.76 (3H, s, OCH_3_), 5.17 (1H, d, *J* = 9 Hz, H-2"), 5.53 (1H, d, *J* = 4.5 Hz, H-2), 5.75 (1H, d, *J* = 2.0 Hz, H-6), 5.84 (1H, d, *J* = 2.0 Hz, H-8), 5.86 (1H, d, *J* = 2.0 Hz, H-6"), 5.97 (1H, d, *J* = 2.0 Hz, H-8"), 6.74~7.16 (8H, m, H-Ar). HR-ESI-MS: *m/z* 557.1446 [M+H]^+^. Spectral data were in accordance with those reported in the literature [4,8], which confirmed that the isolated compound **2** was sikokianin B.

*Sikokianin* A (**3**). Yellow amorphous powder. ^1^H-NMR: δ_H_ 2.91 (1H, d, *J* = 2.0 Hz, H-3), 2.98 (1H, d, *J* = 2.0 Hz, H-3"), 3.82 (3H, s, OCH_3_), 5.32 (1H, d, *J* = 2.0 Hz, H-2), 5.37 (1H, d, *J* = 2.0 Hz, H-2"), 5.75 (2H, d, *J* = 0.5 Hz, H-6, H-6"), 5.88 (2H, d, *J* = 0.5 Hz, H-8, H-8"), 6.63~7.04 (8H, m, H-Ar). HR-ESI-MS: *m/z* 557.1448 [M+H]^+^. Spectral data were in accordance with those reported in the literature [8], which confirmed that the isolated compound **3** was sikokianin A. 

## 4. Conclusions

In conclusion, one new biflavanone, 5,5',7,7'-tetrahydroxy-2-(4-hydroxyphenyl)-2'-(4-methoxy-phenyl)-[3,3'-bichroman]-4,4'-dione (1), together with two known compounds, sikokianin B (2) and sikokianin A (3) was isolated from the EtOH extract of the roots of *Wikstroemia indica*. 
